# Right Ventricular Dysfunction before and after Cardiac Surgery: Prognostic Implications

**DOI:** 10.3390/jcm13061609

**Published:** 2024-03-11

**Authors:** Anna Merlo, Camilla Cirelli, Enrico Vizzardi, Laura Fiorendi, Federica Roncali, Marco Marino, Maurizio Merlo, Michele Senni, Edoardo Sciatti

**Affiliations:** 1 School of Medicine and Surgery, University of Milan-Bicocca, 20126 Milan, Italy; a.merlo@campus.unimib.it (A.M.); c.cirelli1@campus.unimib.it (C.C.); msenni@asst-pg23.it (M.S.); 2 Section of Cardiovascular Disease, Department of Medical and Surgical Specialties, Radiological Sciences and Public Health, University of Brescia, 25133 Brescia, Italy; vizzardi72@gmail.com; 3 Cardiology 1 Unit, ASST Papa Giovanni XXIII, 24121 Bergamo, Italy; laurafiorendi1@gmail.com (L.F.); federica.roncali@gmail.com (F.R.); marcomarino14@gmail.com (M.M.); 4 Cardiac Surgery Unit, ASST Papa Giovanni XXIII, 24121 Bergamo, Italy; mmerlo@asst-pg23.it

**Keywords:** right ventricular dysfunction, cardiac surgery, echocardiography, prognosis

## Abstract

Right ventricular dysfunction is a prognostic factor for morbidity and mortality across a broad spectrum of cardiovascular diseases. While the role of the right ventricle in surgical patients has emerged, the prognostic impact of right ventricular dysfunction remains unclear in a large cardiac surgery population. We reviewed the existing literature about the role of right ventricular dysfunction in adults undergoing different kinds of cardiac surgery either present before or developed after surgery itself. Pre- and post-operative right ventricular dysfunction has demonstrated substantial prognostic implications. However, there remains a lack of consensus regarding its definition and diagnostic criteria. The available literature is limited to small-sized studies, underscoring the need for studies with larger populations.

## 1. Introduction

Right ventricular dysfunction (RVD) is a prognostic factor for morbidity and mortality across a broad spectrum of cardiovascular diseases [1,2]. While the role of the right ventricle (RV) in surgical patients has emerged, the prognostic impact of RV function remains unclear in large cardiac surgery populations.

Post-cardiac surgery involving cardiopulmonary bypass (CPB) and pericardial incision changes RV contractility, showing a decrease in longitudinal shortening and an increase in transverse shortening of the RV even when global function is normal [3]. In certain cases, this can progress to perioperative RV failure (RVF), an infrequent but highly severe condition [4]. 

In this context, a comprehensive evaluation of RV function is crucial, especially in patients undergoing cardiac transplant or left ventricular assist device (LVAD) implantation. However, the literature lacks a clear and unequivocal definition of RVD [5], and studies employ different criteria, including various clinical, echocardiographic, cardiac magnetic resonance (CMR), and hemodynamic parameters.

The aim of this review is to provide an overview of RVD before and after cardiac surgery, encompassing coronary artery bypass grafting (CABG), valvular procedures, heart transplantation, LVAD implantation, and congenital heart surgery, along with an exploration of its prognostic implications.

## 2. Right Ventricular Dysfunction before Cardiac Surgery

In recent years, preoperative RVD has been recognized as a significant risk factor for adverse outcomes in patients undergoing cardiac surgery. The presence of severe RVD has a significant impact on postoperative management; moreover, it is a contraindication for certain cardiac surgeries. In LVAD implantation, assessing RV function is necessary for patient selection and risk stratification, and RVD serves as an exclusion criterion [6]. Additionally, in isolated severe secondary tricuspid regurgitation, surgery is not indicated in patients with severe RVD [7]. However, commonly used preoperative risk scores such as the Society of Thoracic Surgeons (STS) score or the European System for Cardiac Operative Risk Evaluation (EuroSCORE) do not include the evaluation of RV function [8,9].

Preoperative RVD is attributed to several factors, including pulmonary hypertension, coronary artery disease, pulmonary disease, ventricular interdependence, and cardio-pulmonary interactions [10]. This scenario is not uncommon, affecting approximately 20% of patients undergoing left-sided valve surgery [11]. The prevalence of preoperative RVD is influenced by the type of valvular disease; in aortic stenosis, RV function tends to be preserved compared to mitral stenosis [2,12]. Even in patients undergoing heart transplantation, RVD is not infrequent and serves as a marker of advanced heart failure characterized by severe multiorgan failure.

To better assess the function of the RV before cardiac surgery, echocardiography has traditionally been the most accessible method for comprehensive evaluation. Indeed, echocardiographic parameters have emerged as crucial for assessing preoperative RV systolic function, revealing significant prognostic implications. To define RVD, the guidelines of American Society of Echocardiography (ASE) recommend the assessment of at least one of the following parameters: RV fractional area change (RVFAC), Doppler-derived tricuspid lateral annular systolic velocity (S′), tricuspid annular plane systolic excursion (TAPSE), and right myocardial performance index (RMPI) [13]. RVD should be suspected when S′ is <10 cm/s, TAPSE is <16 mm, RVFAC is <35%, or RMPI is >0.55 [13]. 

In a prospective study conducted on patients undergoing left valve surgery, a preoperative RVFAC < 32% or a RMPI > 0.49 were associated with an increase of postoperative adverse outcomes [10]. Maslow et al. showed similar results in patients with severe left ventricular systolic dysfunction undergoing CABG, demonstrating that patients with preoperative RVFAC < 35% have an increased risk for morbidity and mortality [14]. Additionally, in patients undergoing surgical ventricular reconstruction, preoperative TAPSE < 16 mm emerged as an independent predictor of long-term mortality [15].

Nevertheless, ASE guidelines suggest employing multiple measures of RV function for a more comprehensive assessment, aiding in the distinction between normal and abnormal function. RV function parameters are sometimes discrepant, supporting the need for a multiparametric approach when evaluating RV function before cardiac surgery [16]. In a retrospective study by Towheed and colleagues conducted on 269 patients undergoing cardiac surgery, five echocardiographic parameters were considered for assessing RV function: RVFAC, TAPSE, S′, RVMPI, RV dP/dt. At least three abnormal RV parameters demonstrated a strong prediction of adverse outcomes following left-sided valvular surgery, and patients with preoperative RVD had higher 30-day mortality and were at risk of developing multisystem failure/shock [11]. In a prospective study involving 400 patients undergoing cardiac surgery, multivariate analysis revealed that RVFAC, S′, dP/dt, and isovolumic acceleration (IVA) were significantly associated with overall mortality over three years, regardless of the EuroSCORE. In this study, abnormal RVFAC, S′, and IVA specifically correlated to cardiovascular mortality in valve surgery [17]. 

Alongside the parameters of RV systolic function, it is essential to include RV diastolic function in a thorough evaluation of RV function. Abnormal preoperative RV diastolic profiles have been linked to challenges in discontinuing CPB [18]. However, there are limited studies exploring the prognostic implications of RV diastolic dysfunction in cardiac surgery patients. According to ASE guidelines, the most validated parameters of RV diastolic assessment are Doppler velocities of the transtricuspid flow (E, A, and E/A), tissue Doppler velocities of the tricuspid annulus (E′, A′, E′/A′), deceleration time, and isovolumic relaxation time (IVRT) [13]. In a study by Sumin et al., pre-existing RV diastolic dysfunction in patients undergoing CABG with LVEF > 40% was correlated with a higher frequency of postoperative complications. Specifically, the reduction in the E/A ratio was identified as the most efficacious echocardiographic marker for predicting postoperative complications [19]. In another study conducted on 231 patients undergoing CABG with LVEF ≤ 35%, decreased preoperative RV diastolic function was an independent risk factor for early death following the procedure. Particularly, RV Et/Et′ ≥ 10 was significantly associated with early death after CABG [20].

In addition to more traditional parameters, new echocardiographic variables are emerging in the evaluation of RV function, such as longitudinal strain (LS). In a study involving 250 cardiac surgery patients, impaired RV global LS (RV-GLS), defined as >−21%, exhibited a higher postoperative mortality [21]. Additionally, among other RV systolic indices (TAPSE and RVFAC), only RV-GLS showed an association with patient outcomes in a multivariate analysis adjusted for EuroSCORE-II and CPB duration [21]. In addition to deformation imaging, 3D echocardiography is also considered a new method of choice for evaluating RV function. In a previous study conducted on 26 patients undergoing LVAD implantation, 3D echocardiography-derived RV ejection fraction (RVEF) and 3D RV free wall LS (RVFWLS) were associated with RVF and long-term outcomes [22]. 

Although echocardiography remains a frontline method in this setting, the complexity of RV anatomy, compounded by its retrosternal position, poses challenges in assessing RV function. Nevertheless, advanced imaging techniques are increasingly proving to be valuable tools for facilitating precise morpho-functional evaluations. CMR is considered the gold standard in many cardiac pathologies and for the assessment of RV volumes; however, limited data exist in CMR use for evaluation of RVD in cardiac surgery patients. A study conducted by Lella and colleagues on 109 consecutive patients undergoing isolated CABG or valve procedures demonstrated that reduced RVEF (<35%), as measured by CMR, was an independent risk factor for long-term cardiac rehospitalizations and a better predictor of cardiac re-hospitalization compared to reduced LVEF (<45%). In addition, patients who underwent valve surgery with an abnormal RVEF had an increased incidence of late repeat cardiac hospitalizations [23].

In addition to imaging techniques, right heart catheterization (RHC) remains a valid method for the preoperative assessment of RV function. In a study conducted by Boldt and colleagues in patients undergoing aortic valve replacement, RV function was evaluated with RVEF measured by means of the thermodilution technique. This study demonstrated a higher postoperative use of inotrope in patients with RVD [24]. Fiorentino and colleagues analyzed 517 patients who underwent heart transplant between 2000 and 2020. They defined RVD as central venous pressure (CVP) > 15 mmHg and CVP/pulmonary capillary wedge pressure ratio >0.63. This study demonstrated that 1-, 5-, and 10-year survival after heart transplant were significantly worse in patients with preoperative RVD than patients without [25]. This finding was confirmed in another study conducted by Bellettini et al. on 657 patients undergoing heart transplants, showing that preoperative RVD, defined as a PAPi value < 1.68, was associated with mortality and morbidity after heart transplant, providing incremental prognostic value over traditional parameters [26]. Moreover, in a systematic review by Essandoh and colleagues including 4756 patients undergoing LVAD implantation, preoperative PAPi emerged as a clinical predictor of postoperative RVF [27].

In recent times, there has been growing interest in investigating the interactions among different organs. Particularly concerning RVD and congestion, it has been hypothesized that they may influence alterations in the anatomy and stiffness of the liver. A recent study conducted by Zvonimir et al. demonstrated that a higher liver stiffness, assessed by ultrasound elastography, was associated with impaired RV and right atrial function [28]. Furthermore, the role of liver stiffness has proven to be significant in preoperative assessment among cardiac surgery patients. A previous study involving 105 patients undergoing non-emergent CABG demonstrated that preoperative liver stiffness was significantly associated with prolonged postoperative hospital length of stay [29]. In this context, it would be useful to correlate the presence of liver stiffness with the presence of preoperative RVD or the risk of developing RVD postoperatively. However, there are currently a lack of studies exploring this correlation and its potential prognostic implications. The cited studies assessing the prognostic value of RVD before cardiac surgery are listed in Table 1.

## 3. Right Ventricular Dysfunction after Cardiac Surgery

RVD following cardiac surgery represents a well-established contributor to both morbidity and mortality. However, a consensus on the precise definition of RVD and RVF, as well as standardized diagnostic criteria, remains elusive. RVF is the extreme of the RVD spectrum and can be better defined as “inability of RV to maintain enough blood flow through pulmonary vasculature to achieve adequate LV filling” [30,31]. During cardiac surgery, acute RVD arises as a result of either excessive volume or pressure overload, altered ventricular interdependence, or intrinsic myocardial contractile dysfunction that can be caused by hypoxia or myocardial ischemia, microemboli, air emboli, arrhythmias, long CPB time, reperfusion lung injury with secondary pulmonary hypertension (PH), preexisting pulmonary vascular disease, or sepsis-associated myocardial depression. Acute RVF can ultimately result in systemic congestion and circulatory failure [31]. RVF is reported to occur in up to 0.1% of patients following cardiotomy, in 2–3% of patients following heart transplantation, and in 20–30% of patients after LVAD implantation [32]. In a recent cohort study by Levy et al., RVF was reported in 2.9% of the 3826 patients who underwent cardiac surgery with CPB [33]. 

Over the years and across various studies, different methods have been used to assess RV systolic function, including invasive techniques such as pulmonary artery catheterization and imaging modalities such as echocardiography and magnetic resonance. The gold standard for assessing right ventricular function is echocardiography. If available, 3D echocardiography is recommended to assess RV size and function. Echocardiography can help determine the cause of RV dysfunction. As reported by Petrun and colleagues, in cases of systolic RVD, there will be TAPSE < 17 mm, RVFAC < 35%, or RV S′ < 10 cm/s. In cases of right-sided pressure/volume overload there will be RV basal end-diastolic diameter > 41 mm, RV/LV basal end-diastolic diameter ratio > 1.0, septal shift, or D-shaped LV, RV thickness > 5 mm, inferior vena cava diameter > 21 mm and collapsibility < 50%, and TR peak systolic velocity > 2.8 m/s. In cases of isolated diastolic LV D-shaping, the RV experiences volume overload. In cases of RV increases after load, D-shaping is present during the whole cardiac cycle [30]. More recently, the strain tracking technique has also been introduced in echocardiographic evaluation, allowing for the quantification of myocardial systolic deformation (known as strain and strain rate). 

As previously mentioned, the development of right ventricle dysfunction in the post-cardiac surgery setting has important implications for the patient’s prognosis. According to a retrospective analysis conducted by Bootsma et al., a significant association was identified between postoperative RVEF and long-term mortality in a cohort comprising 3094 heterogeneous post-cardiac surgery patients. Furthermore, the impact on mortality was not limited to the perioperative period but persisted, exacerbating outcomes in the years following the intervention [34].

A study conducted by Diller and colleagues demonstrated a decrease in right ventricular function following coronary artery bypass surgery, with only partial recovery observed over an 18-month follow-up period. Furthermore, there were no differences observed between patients undergoing off-pump or on-pump surgery [35]. 

Concerning mitral valve surgery, numerous studies over the years have shown the occurrence of both temporary and persistent post-surgical RVD. Orde et al. demonstrated that post-mitral valve repair surgery, RVD, is common. Notably, they observed a significantly smaller deterioration in RV function and a more pronounced recovery within the minimally invasive surgery cohort as opposed to the group undergoing standard open sternotomy mitral valve surgery [36]. In a prospective study of degenerative mitral valve disease by Grapsa et al., it has been demonstrated that mitral valve repair leads to more favorable RV remodeling compared to valve replacement [37].

The PREPARE-MVR study, utilizing 3D echocardiography, demonstrated that mitral valve replacement (MVR) induces a significant shift in the RV mechanical pattern. Indeed, pre-operative alterations attributable to mitral regurgitation render the RV susceptible to developing overt dysfunction during and immediately after open-heart surgery. This study indicated that radial motion could effectively compensate for the post-operative decline in longitudinal motion, maintaining RVEF. Interestingly, six months after the successful operation, the native contraction pattern is restored, with RV longitudinal and radial contributions normalized. However, M-mode and 2D indices of longitudinal RV function still indicate incomplete recovery [38].

Lastly, another noteworthy complication observed in patients who underwent mitral valve repair surgery is systolic anterior motion (SAM). The incidence of SAM after mitral valve repair typically falls between 5 to 10%. SAM can manifest particularly in the presence of hypervolemia. Excessive fluid levels during both the perioperative and postoperative phases can lead to a leftward shift of the septum. Additionally, increased right ventricular volume, often indicated by elevated pulmonary artery pressures, can further contribute to bulging the ventricular septum leftward, consequently narrowing the left ventricular outflow tract (LVOT) [39]. In such situations, the immediate consideration of nitroglycerin to reduce pulmonary artery pressures is advisable (SAM: systolic anterior motion of the anterior mitral valve leaflet post-surgical mitral valve repair) [39].

Consistent with previously mentioned findings regarding the mitral valve, Hashemi and colleagues observed a lesser reduction in RV function in patients undergoing minimally invasive aortic valve replacement surgery (MIAVR) compared to those undergoing conventional aortic valve replacement surgery (AVR), despite longer CPB time in the MIAVR group. Moreover, intrinsic RV contractility quantified by strain rate was preserved following MIAVR, while it was deteriorated following AVR [40]. 

In an interesting prospective study by Kammerlander et al. on 539 patients with previous left heart valve procedure, RVD was independently associated with overall mortality, whilst tricuspid regurgitation (TR) was not. The development of TR after heart valve procedures is a consequence of alterations in RV geometry secondary to post-capillary pulmonary hypertension (PH) associated with the left heart valve abnormality. PH induces RV pressure overload, RV dilatation, distortion of the tricuspid valvular apparatus, and finally, TR. However, from the results of this study, it emerges that correcting TR may not be sufficient to modify the patient’s outcome [41]. 

LVAD implantation is complicated by RVF in up to 20% of patients in the early 30-day post-LVAD period [42]. RVF is a concerning complication that can result in compromised LVAD flow, challenges in weaning from CPB, diminished tissue perfusion, multi-organ failure, and a 20% decrease in one-year survival [43]. The key pathophysiological factors of RVD appear to involve the acute unloading of the left heart and the increasing venous return, which may potentially overwhelm a functionally impaired right RV, resulting in RV dilatation, TR, and a leftward shift of the interventricular septum. Since the RV relies significantly on the left ventricle (LV) for its contractile function, the leftward shift of the septum has the potential to alter the geometry of the right ventricle and influence its contractility [31,44]. Risk stratification of patients undergoing LVAD implantation is crucial for identifying candidates who might require RV support, enabling timely pharmacological intervention and ultimately improving patient outcomes. From this perspective, the EUROMACS-RHF risk score has been developed and validated. It utilizes a straightforward five-item scoring system to predict early RHF following LVAD implantation. The EUROMACS-RHF risk score is composed of severe RV dysfunction, ratio RA/PCWP ≥ 0.54, advanced INTERMACS class 1 through 3, need for ≥3 intravenous inotropes, and hemoglobin ≤ 10 g/dL. A patient with a high-risk score may necessitate preoperative optimization of RV support, consideration for a biventricular assistance device, or even total heart support [42].

Late RHF is a relatively common and persistent morbidity following continuous-flow LVAD implantation [43]. While late RHF does not impact survival during LVAD support, its occurrence is linked to poorer overall outcomes. It can be primarily associated with intrinsic right ventricular myocardial disease or may be secondary to various causes including ventricular arrhythmia, progression of tricuspid regurgitation, and pulmonary hypertension. Takeda et al. found that diabetes mellitus, BMI > 29, and a blood urea nitrogen level >41 mg/dL were significant predictors for late RHF. Identifying these risk factors is clinically relevant because planned biventricular assist device implantation may result in better outcomes [45].

The incidence of RVD following heart transplantation can vary depending on various factors, including the patient’s conditions, the quality of the transplanted organ, and the surgical procedures employed. RHF after heart transplantation is one of the most important causes of death in the early postoperative course, and it is associated with a significantly increased incidence of complications [46]. Several factors contribute to the development of RVD of the graft, including pulmonary hypertension, suboptimal organ preservation, prolonged ischemic time, mechanical obstruction at the pulmonary artery anastomosis, significant donor–recipient mismatch (particularly when there is more than a 20% mismatch in size), and acute allograft rejection [31]. The cited studies assessing the incidence and the prognostic value of RVD after cardiac surgery are reported in Table 2.

## 4. Right Ventricular Dysfunction and Congenital Heart Disease Surgery

Right heart dysfunction is strongly associated with unfavorable clinical outcomes in congenital heart disease. In this extremely diverse population, the RV assumes a pivotal role in disease progression and prognosis. This holds true whether the RV serves as the subpulmonary ventricular (i.e., atrial septal defects, tetralogy of Fallot, Ebstein’s anomaly, and pulmonary stenosis) or as the systemic ventricle. (i.e., congenitally corrected transposition of great arteries and hypoplastic left heart syndrome with Fontan palliation) [47]. In this setting, the pathophysiological relation between RV function and the underlying disease is extremely complex and goes beyond the aim of this article. However, it has been shown that patients with congenital heart disease experience a reduction in RV function immediately following cardiac surgery. In a study by Shuuring and colleagues, decline in RVF was equal in right-, left- and both-sided surgeries. Despite a gradual improvement over time, complete recovery was not observed 18 months post surgery [48]. In a subsequent study, it was demonstrated that the strongest determinants of RVD after cardiac surgery were preoperative impaired RV function, supraventricular tachycardia, and CPB time > 150 min [49]. Regarding the assessment of RV function, the same considerations made for adult cardiac surgery apply to congenital heart surgery. Longitudinal parameters measuring the systolic function of the right ventricle (such as TAPSE, tissue Doppler imaging, and RV global longitudinal peak systolic strain) are influenced by changes in load and do not show a consistent relationship with RVEF. This lack of correlation is due to regional geometric changes that occur after cardiac surgery, as well as alterations in the pattern of RV contraction. Instead, FAC serves as a reliable proxy for assessing global systolic function and exhibits the strongest correlation with RVEF as measured by MRI. Therefore, FAC should also be preferred for ongoing clinical evaluations over longitudinal parameters after congenital heart surgery [50].

## 5. Unmet Needs and Future Perspectives

**Figure 1:**
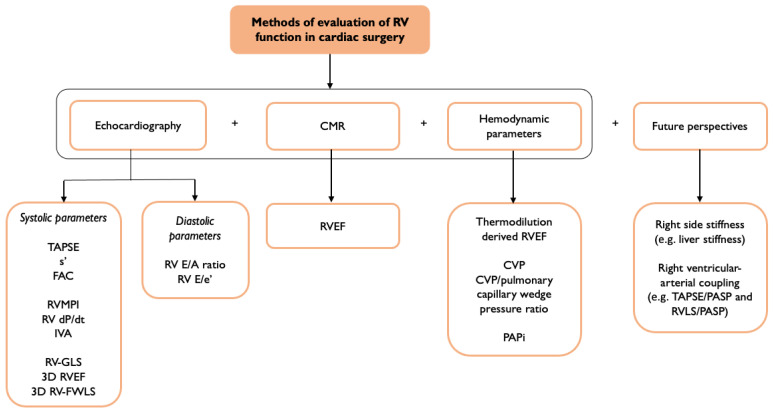
Current and future methods of evaluation of right ventricular function [10,11,14,15,17,19,20,21,22,23,24,25,26,34,35,41].

The assessment of RV function is deemed necessary based on the existing literature regarding cardiac surgery patients; however, there are still gaps in the evidence from this setting. Primarily, there are a lack of studies involving larger populations that could confirm the prognostic importance of RVD both before and after cardiac surgery, as is suggested by the current data. Moreover, regarding the preoperative study of RV function, we should move beyond the evaluation of simple RV parameters such as TAPSE and FAC. A comprehensive overview of how we might image RV function is displayed in Figure 1.

Although MRI remains the gold standard for estimating the RVEF, its accessibility is limited, and 3D echocardiography could potentially serve as a more accessible method, allowing for a comprehensive assessment of RV function in this setting. However, concerning both before and after cardiac surgery, there are limited data focusing on the use of 3D for study of RVD by transesophageal echocardiography in the operating room before sternotomy; additionally, these data were estimated during general anesthesia and assisted ventilation and may not be very reliable [22].

Furthermore, evaluation of RVD should also encompass data on right ventricular–arterial coupling, which has shown significant prognostic implications in patients undergoing transcatheter procedures [51,52]. In a retrospective study involving 56 patients undergoing trans-catheter aortic valve implantation (TAVI), preoperative deformation imaging (RVLS) and right ventricular–arterial coupling (estimated by TAPSE/PASP and RVLS/PASP) provided stronger prognostic implications than other RV echocardiographic parameters [51]. These findings were further confirmed in a cohort of 226 patients undergoing MitraClip implantation, wherein TAPSE/PASP demonstrated better prognostic value compared to either TAPSE or PASP individually [52]. Additionally, a study from Adamo et al., conducted on 501 patients undergoing transcatheter edge-to-edge mitral valve repair (M-TEER) showed that an improvement in TAPSE/PASP after the procedure was independently associated with reduced risk of mortality upon long-term follow-up [53].

Despite these results from percutaneous procedures, there are no data on the assessment of right ventricular–arterial coupling by echocardiography before and after cardiac surgery, which could be a future research direction to pursue.

## 6. Conclusions

Pre- and post-operative RVD has demonstrated substantial prognostic implications. However, there remains a lack of consensus regarding its definition and diagnostic criteria. A thorough assessment of RV function has become essential in cardiac surgery patients, particularly before and after procedures such as LVAD implantation or heart transplantation. Nevertheless, the available literature is limited to small-sized studies, underscoring the need for studies with larger populations.

## Figures and Tables

**Table 1:** Selected studies assessing the prognostic value of RVD before cardiac surgery.

Authors, Year	Sample Size and Study Design	Population	RVD Criteria	Main Findings
Maslow et al., 2002 [14]	41 (retrospective)	CABG with LVEF < 25%	RVFAC < 35%	RVD is associated with lower long-term survival
Haddad et al., 2007 [10]	50 (prospective)	High risk valvular surgery	RVFAC < 32% or RVMPI > 0.49	Higher incidence of postoperative circulatory failure in preoperative RVD
Ternacle J et al., 2013 [21]	250 (prospective)	CABG (50.4%), valve surgery (49.6%)	RV-GLS > −21%,	Impaired RV-GLS had higher postoperative death (22% vs. 3%)
Garatti et al., 2014 [15]	324 (retrospective)	Ischaemic cardiomyopathy submitted to surgical ventricular reconstruction	TAPSE < 16 mm	5- and 8-year survival rate and freedom from cardiac events were significantly lower in patients with RVD
Lella LK et al., 2015 [23]	109 (retrospective)	CABG (56%), valve surgery (44%)	RVEF < 35%	Higher incidence of long-term cardiac re-hospitalization in RVD
Peyrou J et al., 2017 [17]	400 (prospective)	CABG (49%), valve surgery (63%), CABG + valve surgery (12.7%)	At least one parameter among RVFAC < 35%, S′ < 10 cm/s, RVMPI > 0.55, RV dP/dt < 400 mmHg/s, GLS > −17% and IVA < 1.8 m/s^2^	In the CABG subgroup, RVFAC < 35% and S′ < 10 cm/s were predictive of overall mortality; in the valve subgroup, RVFAC < 35%, S′ < 10 cm/s and IVA < 1.8 m/s^2^ were predictive of overall mortality.
Magunia et al., 2018 [22]	26 (retrospective)	LVAD	Impaired 3D RVEF and 3D RVFWLS	Lower 3D RVEF and 3D RV FWLS are associated with right ventricular failure and long-term outcome
Towheed A et al., 2021 [11]	359 (retrospective)	Left valve surgery	At least 3 abnormal RV parameters of 5 including RVFAC, TAPSE, S′, RVMPI, and RV dP/dt	Higher 30-day mortality (RVD 22.6% versus 3.8%)
Bellettini M et al., 2022 [26]	657 (retrospective)	Heart transplantation	PAPi < 1.68	Lower 1-year survival rates post HTx in patients with preoperative RVD
Fiorentino M et al., 2023 [25]	517 (retrospective)	Heart transplantation	CVP > 15 mmHg and CVP/pulmonary capillary wedge pressure ratio >0.63	Lower 1-, 5-, 10- year survival rate post HTx in patients with preoperative RVD

**Table 2:** Selected studies assessing the incidence and the prognostic value of RVD after cardiac surgery.

Authors, Year	Population	Follow-Up	Surgery	RVD Criteria	Main Findings
Klima et al., 2005 [46]	591		Heart transplantation (HT)	Necessity of postoperative IABP, ECMO, RVAD or ballooned RV + end organ failure of liver/kidney/intestine	RVF contributes by 13.2% to all deaths after HT Duration of stay in ICU and duration of mechanical ventilation was prolonged in patients with RHF
Diller et al., 2008 [35]	32	18 months	CABG	Reductions in s′ and E′ values	RV function decreases after CABG with only incomplete recovery over time
Kormos et al., 2010 [43]	484		LVAD	Necessity of RVAD implantation, >13 days of inotropic support, inotropic support starting > 14 days after implantation	Patients with RVF had significantly worse survival and longer hospitalization time before discharge
Kammerlander et al., 2014 [41]	539	53 ± 15 months	Left heart valve procedure	FAC < 35%, TR severity	RVD, but not TR, is independently associated with survival late after left heart valve procedures
Bootsma et al., 2017 [34]	1109	4 years	CABG, valve surgery	Thermodilution-derived RVEF	RV function is independently associated with 2-year all-cause mortality
Soliman et al., 2018 [42]	2000	2 years	LVAD	Need for postoperative mechanical RV support, need for prolonged postoperative inotropic support and need for prolonged NO ventilation	Early severe RVF occurs in 21.7% of patients with LVAD and is associated with high mortality (up to 29%).

## Data Availability

Not applicable.
